# Slowdown of Water Dynamics from the Top to the Bottom of the GroEL Cavity

**DOI:** 10.1021/acs.jpclett.1c01216

**Published:** 2021-06-15

**Authors:** Nicolas Macro, Long Chen, Yushan Yang, Tridib Mondal, Lijuan Wang, Amnon Horovitz, Dongping Zhong

**Affiliations:** † Department of Physics, The Ohio State University, Columbus, Ohio 43210, United States; ‡ Department of Structural Biology, Weizmann Institute of Science, Rehovot 76100, Israel; § Department of Chemistry and Biochemistry, Programs of Biophysics, Program of Chemical Physics, and Program of Biochemistry, The Ohio State University, Columbus, Ohio 43210, United States

## Abstract

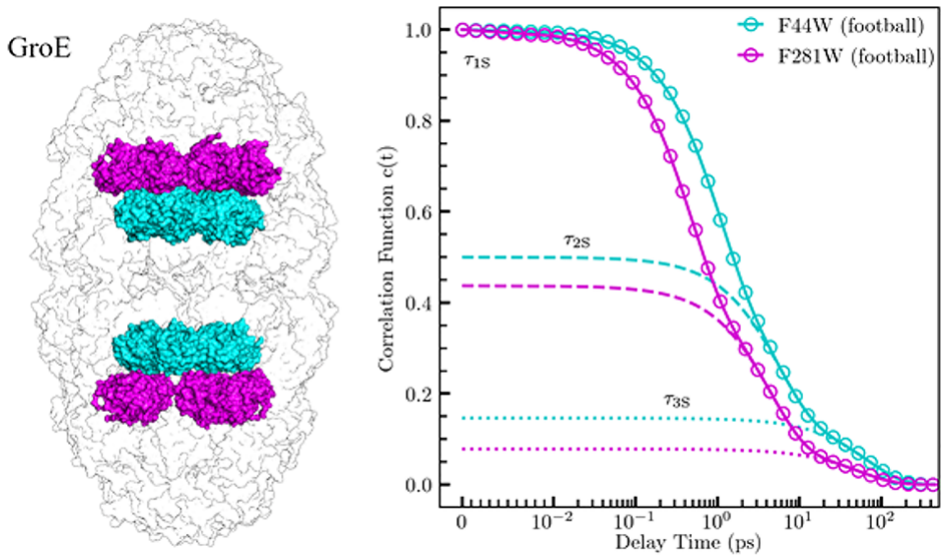

The GroE molecular chaperone system is a critical protein machine that assists the folding of substrate proteins in its cavity. Water in the cavity is suspected to play a role in substrate protein folding, but the mechanism is currently unknown. Herein, we report measurements of water dynamics in the equatorial and apical domains of the GroEL cavity in the apo and football states, using site-specific tryptophanyl mutagenesis as an intrinsic optical probe with femtosecond resolution combined with molecular dynamics simulations. We observed clearly different water dynamics in the two domains with a slowdown of the cavity water from the apical to equatorial region in the football state. The results suggest that the GroEL cavity provides a unique water environment that may facilitate substrate protein folding.

Molecular chaperones, which are found across biology, prevent aggregation and facilitate protein folding.^1^ The GroE system from *Escherichia coli* is an ATP-dependent protein folding facilitator both *in vitro* and *in vivo* where it assists in the folding of ∼250 proteins and is required for the proper folding of ∼60 proteins.^2−5^ The GroE system is composed of two proteins: GroEL and its helper-protein GroES (Figure 1). GroEL consists of 14 identical 56 kDa subunits that form two 7-member rings that are placed back-to-back, with cavities at each end.^6^ The helper-protein GroES is a homoheptamer consisting of 10 kDa subunits that form a single ring. ATP-dependent binding of GroES to the apical domains of GroEL leads to encapsulation of the substrate protein in the cavity.^7^ There are currently many models that describe the ATP- and substrate-dependent reaction cycle of GroEL^8−10^ which is governed by cooperativity between GroEL monomers.^3^ There is positive intraring cooperativity and negative inter-ring cooperativity in ATP binding producing two GroEL-GroES complexes that are both functional: the asymmetric GroEL_14_:GroES_7_ (bullet) and the symmetric GroEL_14_:GroES_14_ (football) complexes^10^ (see Figure 1). The mechanism by which GroEL facilitates substrate folding is hotly debated with many models requiring experimental validation. There are two main categories of models describing the GroEL substrate folding mechanism, named the passive cage and the active model.^11−17^ The passive cage model states that GroEL does not alter the folding pathway of the substrate and merely provides an environment for the normal pathway to occur.^11,12^ The active model assumes there are some interactions between GroEL and the substrate that affect and sometimes enhance the folding process.^13−17^ There are many factors that can affect substrate folding, such as cavity-wall chemical identity, steric confinement, and cavity water properties. Theoretical examinations have provided analysis that suggests the confinement of solvent could result in improved folding rate.^15,18^ Alternatively, increased rigidity of water motions would promote protein unfolding and assist in an annealing mechanism.^17,19^

**Figure 1:**
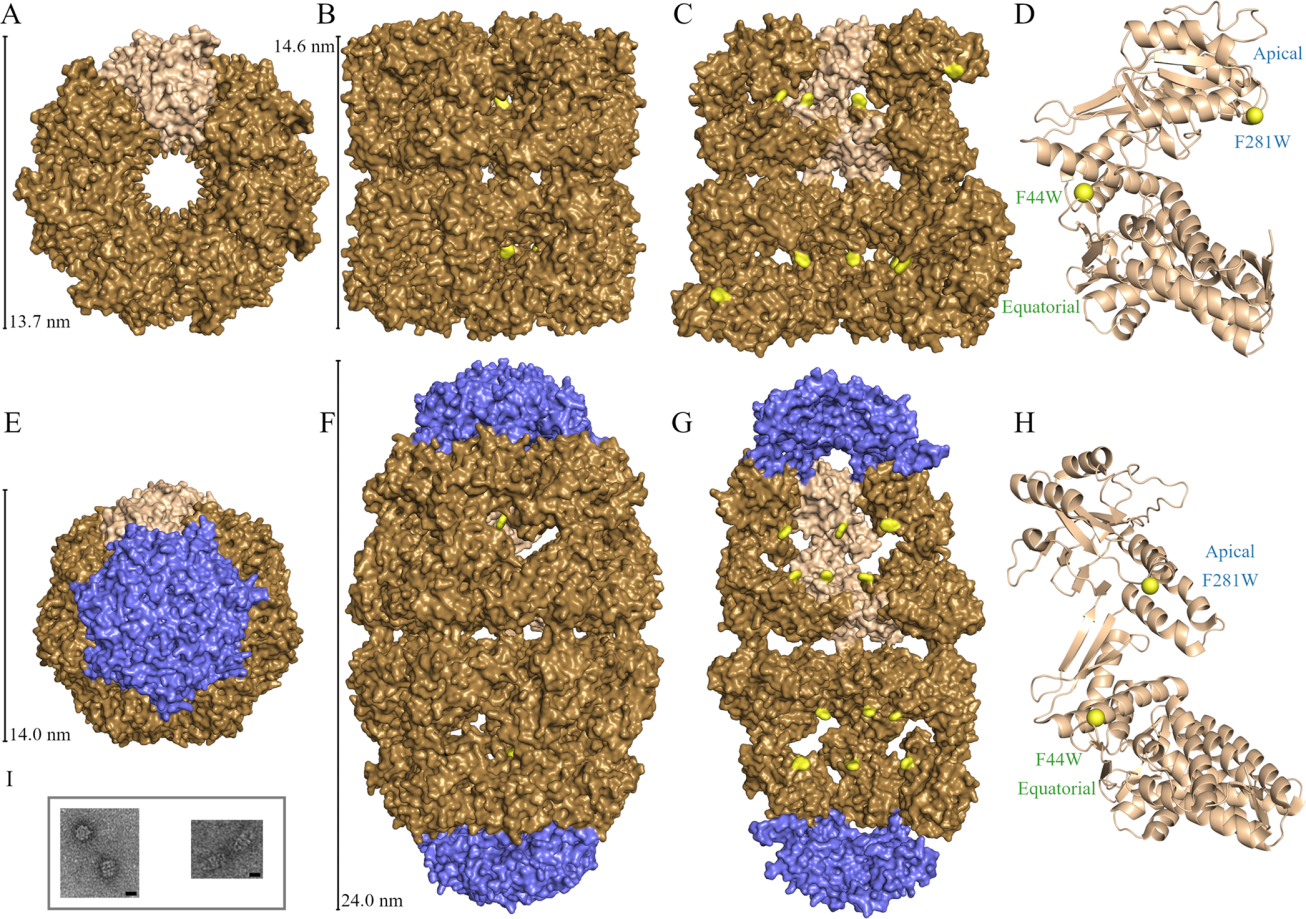
Crystal structure of GroEL apo (top row [A–D], PDB ID: 5W0S) and football (bottom row [E–H], PDB ID: 4PKO) states. Both mutations are represented in yellow, with GroES represented as blue. The structures are viewed from the top (first column [A and E]), side (second column [B and F]), sector view of interior (third column [C and G]), and monomer ribbon with mutation sites at yellow spheres (fourth column [D and H]). Panel I contains representative examples of TEM images taken to verify apo and football states. Scale bar is 10 nm.

Water is a critical solvent for cytosolic proteins to fold into their native state and to function.^20−24^ Water in the hydration layers near a protein surface has been observed to have significantly slower dynamics than that of bulk water.^25^ Many previous studies have been performed to understand the hydration dynamics of water near the protein surface, including NMR,^26^ neutron scattering,^27^ 2D-IR,^28^ THz absorption spectroscopy,^22,29^ and molecular dynamics (MD) simulations.^30^ These studies have determined that these hydration layers have dynamics that are significantly slowed compared to bulk-water dynamics. Additionally, just as the surface of proteins is highly heterogeneous with various charged, polar, and hydrophobic residues, the water dynamics across the surface is also highly heterogeneous. A previous study measured the dynamics of water near the apical domains of the GroEL cavity and found the properties of water in the probed region to be similar to those of bulk water.^31^

We developed an ultrafast spectroscopic methodology to measure the dynamics of hydration water around proteins with single-site specificity.^32^ Site-directed tryptophanyl mutagenesis combined with femtosecond fluorescence spectroscopy allows for the detection of dynamics with single-site spatial and femtosecond temporal resolution. This technique has been applied to a wide variety of biological systems, including both α-helical and β-sheet proteins,^33−35^ the enzymatic active site of DNA polymerase IV,^36,37^ and the confined environment of lipidic cubic phase.^38^ Here, both GroEL and GroES do not contain single tryptophanyl residue; thus, we use the same strategy and report on measurements for two mutations F44W and F281W, one at a time, in both the apo (GroEL_14_) and football (GroEL_14_:GroES_14_) states (Figure 1). We did not carry out measurements for the asymmetric GroEL_14_:GroES_7_ bullet state because the tryptophan probes in the two rings of this state are in potentially different environments. The choice of the two mutants is based on residues that are not involved in contacts, are not in the middle position of α-helices or β-strands, and are not coupled with other residues, and actually the two mutants are located from the top to the bottom of the cavity. Our measurements enable us to detect the changes in hydration dynamics inside the GroEL cavity in the apical (F281W) and equatorial (F44W) domains, which span the entire region where substrate protein has been found to reside.^39^

We measured nine femtosecond-resolved fluorescence transients from the blue (310 nm) to red (370 nm) side of the emission peak (λ_peak_) with a time window of 3 ns for each mutation site in the apo and football states (Figure 2A,B). These transients detect both the solvation and lifetime processes. The solvation processes show typical decays at the blue side and rises at the red side of the emission peaks with lifetime decays across all wavelengths. For apo F44W, we observed three solvation processes in 0.3–1.3, 1.5–11, and 20–59 ps along with two lifetime decays at 315 ps and 3.6 ns. In the F44W football state, we detected three similar solvation dynamics in 0.3–1.1, 1.6–8.6, and 30–70 ps with two lifetimes of 271 ps and 2.3 ns. The steady-state emission peaks of both states are around 350 nm, thereby indicating that F44W is exposed to cavity water near the equatorial wall with a significant number of water molecules near the probe. In the case of apo F281W, we observed three solvation processes in 0.3–0.6, 1.2–8.8, and 20–49 ps with two lifetimes of 240 ps and 1.6 ns. In the football state with F281W, the solvation processes were observed to occur in 0.2–0.5, 1.5–6.9, and 20–59 ps with two lifetime decays at 284 ps and 1.8 ns. The steady-state emission peak is 339 nm for the apo state, suggesting that the probe is almost fully inserted into the surface, whereas it is 350 nm for the football state, indicating that the probe detects a significant number of water molecules near the cavity wall of the apical domain in this state. Clearly, structural changes occur from the apo to football state, enabling the probe to detect more water molecules in the latter state. We also note that each of the sites exhibits a subnanosecond lifetime decay which can potentially merge with the longest solvation process in tens of picoseconds. We suspect these shorter lifetimes are due to quenching from nearby residues.^40^ This quenching does limit the efficacy of the measurement in that it may hinder the detection of the slowest third solvation relaxation dynamics.

**Figure 2:**
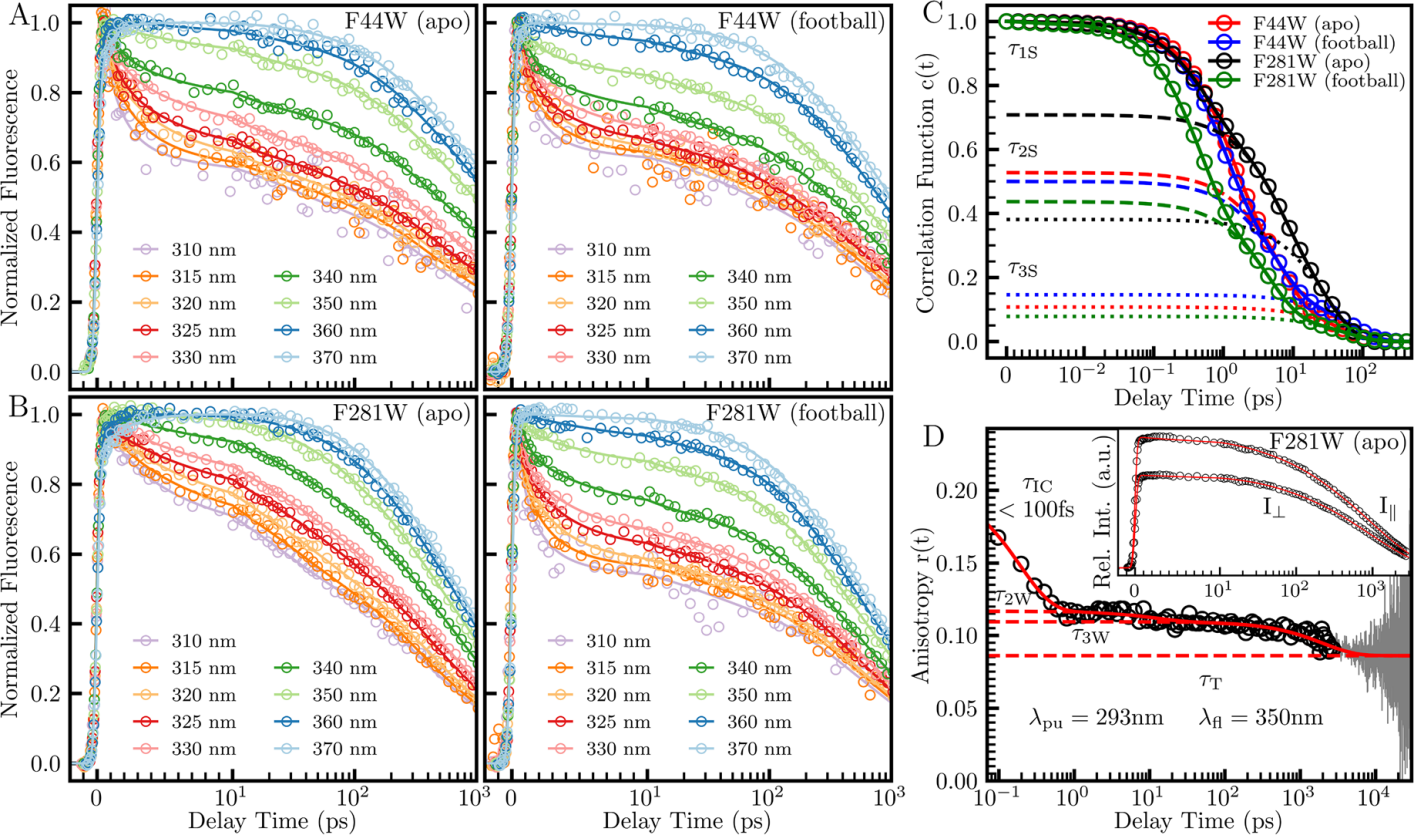
Normalized fluorescence transients with correlation functions and example of anisotropy transients with calculated anisotropy. (A and B) Normalized fluorescence transient data (circles) with fit lines for the mutations F44W and F281W in each state. (C) Solvation correlation functions calculated from the transients of A and B. The dashed and dotted lines represent components of the correlation functions. (D) Apo F281W upconversion anisotropy data (circles), long-time TCSPC data (gray), and fit line (red) with associated upconversion transients in the inset.

Using the strategy that we recently developed,^32^ we constructed femtosecond-resolved emission spectra (FRES) and calculated the solvation correlation functions *c*(*t*) shown in Figure 2C and Table 1. The correlation function is fit using a multiexponential decay with an amplitude indicating the percentage of the total Stokes shift associated with a specific process. For apo F44W, we obtained three solvation times of 1.1 ps (47% of total Stokes shift), 6.0 ps (42%), and 51 ps (11%). Football F44W has three solvation times of 0.91 ps (50%), 5.4 ps (35%), and 67 ps (15%). The initial decays in 1.1 and 0.91 ps for the apo and football states, respectively, are significantly slower than the typical value around 0.5 ps,^34−37^ which is usually associated with bulk-type water motions in the outer layers of the hydration shell which are farther than 7 Å from the protein surface. The second component is also relatively longer than the typical value of a few picoseconds. This time increase is associated with a charged environment or a concave geometry^34−37^ and typically represents the collective motions of the inner-layer interfacial water. The third solvation time in tens of picoseconds is in the typical range and usually reflects the subsequent cooperative water-network restructuring dynamics coupled with local protein fluctuations, although the values are likely the lowest limit because of fast quenching. In the case of apo F281W, we obtained three solvation times of 0.51 ps (29%), 5.8 ps (33%), and 30 ps (38%). The football F281W has three similar solvation times of 0.44 ps (56%), 4.4 ps (36%), and 54 ps (8%) but with different amplitudes of the first and third components. Given the different emission peaks and positions, it appears that the F281W site in the apo state is barely exposed and detects fewer ultrafast water molecules in the outer hydration layers while in the football state it is fully exposed to the cavity water and probes more bulk-type water molecules. Similarly, the second and third components show dynamics comparable to those seen for F44W, reflecting the similar nature of these surface water–protein coupled relaxations. All these dynamics are closely correlated with local chemical identities and structural geometries of the protein, as discussed below.

**Table 1:** Time Constants and Speeds for Solvation and Anisotropy Dynamics.^a^

mutant	state	λ_peak_	τ_S1_	τ_S2_	τ_S3_	*S*_1_	*S*_2_	*S*_3_	τ_W2_	τ_W3_	ω_2_	ω_3_
F44W	apo	349.6	1.1	6.0	51	483	75	2.3	27	699	0.71	0.0269
F44W	football	349.5	0.91	5.4	67	525	62	2.1	21	904	1.03	0.0237
F281W	apo	338.4	0.51	5.8	30	543	52	12.0	12	1997	0.81	0.0077
F281W	football	350.2	0.44	4.4	54	1515	95	1.7	11	524	1.57	0.0392

a λ_peak_, steady-state emission peak (nm); τ_S_, solvation correlation function decay time (ps); *S*, solvation correlation function speed (cm^–1^/ps); τ_W_, anisotropy decay time (ps); ω, anisotropy speed (deg/ps).

We performed MD simulations for each mutant in both the apo and football states with a total trajectory time of 3 ns. Snapshots of the last 2 ns are shown in Figure 3 for water molecules within 10 Å from the tryptophan indole ring. Among these water molecules, we further examined the water numbers within 5 Å of tryptophan and the protein surface as well as 7 Å of the protein surface. For apo F44W on a convex protrusion inside an open cave surrounded by the protein surface, its emission peak is at 350 nm, similar to that of fully exposed tryptophan at the protein surface or tryptophan in bulk water. However, we found that almost all of the water molecules are within 7 Å of the protein surface; only about 8 water molecules out of about 170 total water molecules are beyond 7 Å from the protein surface, and 125 waters are within 5 Å of the protein surface (Figure 3). Thus, these water molecules are actually trapped in the cave near the protein surface, and their initial dynamics are mainly due to the local collective water-network relaxations. Considering our previous studies of hydration water on the protein surface,^34−37^ their dynamics would not occur in femtoseconds but mostly in a few picoseconds. Here, we observed for F44W in the apo state two relaxation components of 1.1 ps (47%) and 6.0 (42%) ps, *i.e.*, two very heterogeneous dynamics of around a few picoseconds of 170 water molecules in the cave. In football F44W, there is a significant structural change of the surrounding protein moving farther away from the tryptophan, causing the probe to become fully exposed to the cavity water at the inner protein surface with an emission peak at 350 nm, and thus, we directly detect the cavity-water dynamics. We observed that the initial relaxation occurs in 0.91 ps, *i.e*., roughly two times slower than that of the previously measured outer hydration layers beyond 7 Å from the protein surface.^34−37^ We found that there are about 180 water molecules around the tryptophan, typical of a fully exposed probe at the protein surface, and 40 water molecules are from the outer layers (Figure 3). On a typical protein surface,^34−37^ the outer-layer water is of bulk type and the dynamics occurs in hundreds of femtoseconds. Here, we observed significantly slower dynamics of the initial component in 0.91 ps (50%), indicating that the water molecules in the equatorial domain are less flexible than those in the outer hydration layers of a typical protein surface. This observation can be significant; the water slowdown shows a more rigid water structure and may represent certain alignment in the equatorial domain, as we also observed that the time-zero emission spectrum shifts to the red side with an emission peak of longer than 330 nm, *i.e*., more stabilization energy by the favorable alignment. This probe position is unique, and in the apo and football states, we separately detected the trapped water dynamics in the cave and the cavity-water slowdown near the equatorial surface within 7–10 Å from the protein surface, respectively. The local collective relaxation of the surface water in the inner hydration layers is in 5.4 ps (35%) for football F44W. For both states, the cooperative long-time rearrangements with the local protein occur in tens of picoseconds, similar to the relaxations of water in the inner hydration layers near the protein surface as observed before.^34−37^

**Figure 3:**
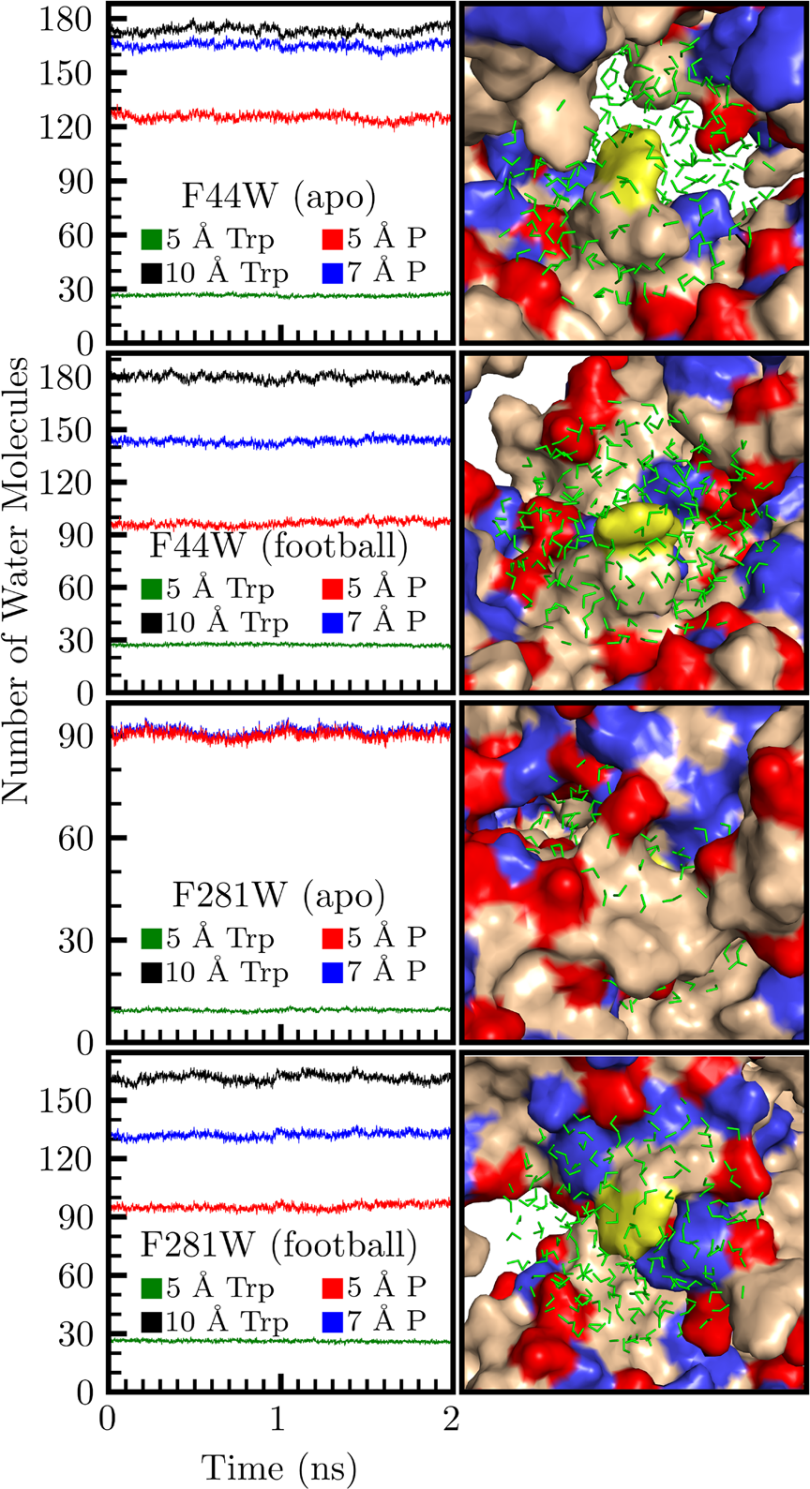
Two nanosecond snapshots of an MD-simulation trajectory. The left column contains the number of water molecules within 10 Å of the tryptophan probe (black) and various subpopulations, averaged across all 14 monomer sites. The water within 5 and 7 Å from any protein surface are in red and blue, respectively. The water 5 Å from the indole ring is labeled in green. The right column contains the local environment around the tryptophan probe (yellow) with positive and negative side chains colored as blue and red, respectively. Water molecules within 10 Å of the indole ring are shown.

Distinct dynamics were also observed for the mutant F281W in the apical domain. In the F281W apo state, the emission peak is 339 nm, indicating that the tryptophan is almost fully inserted into the GroEL exterior facing surface. We observed initial ultrafast dynamics in 0.51 ps (29%), very similar to bulk-type water motions in the outer hydration layers.^34−37^ However, as seen in Figure 3, we see that all 90 water molecules near the tryptophan are within 5 Å of the protein surface, and the relaxation was expected to be within a few picoseconds. The probe position is located in a concave geometry with many charged residues around the tryptophan. Thus, we believe that the ultrafast dynamics must be from the frustrated motions of surface water confined in a charged nanospace. Such frustrated motions can be ultrafast reorientations induced by the surrounding charge fluctuations which are not obviously observed for surface water, as pointed out by early MD simulations.^41,42^ We have also observed similar dynamic behaviors in the position of Y56W in γM7-Crystallin^35^ and Y12W in Dpo4;^36^ both positions are in concave regions as observed here. These confined water molecules can experience ultrafast frustrated motions following the charge fluctuations. The structure significantly changes from the apo to the football state (Figure 1) and the probe becomes fully exposed to the cavity with an emission peak shifting to 350 nm. We observed a typical ultrafast motion in 0.44 ps (56%) with more than 30 water molecules in the outer hydration layers 7–10 Å from the cavity surface (Figure 3). The local collective relations are in a few picoseconds, 5.8 ps (33%) for the apo and 4.4 ps (36%) for the football state, as typically observed on protein surfaces.^34−37^ The correlated long-time rearrangements with the local protein are as usual within tens of picoseconds (Table 1).

Here, we observed different initial water dynamics for the two positions in the two reaction cycle states. In such a large chaperone complex, the water structures and motions in various positions exhibit different behaviors and features. The four systems provide four different structural architectures and local chemical identities. In the equatorial domain, we observed the initial heterogeneous dynamics of 170 surface water molecules trapped in an open cave in the apo state and the slowdown of 40 cavity-water molecules by a factor of 2 in the football state. In the apical domain, we found the frustrated ultrafast motions of 90 surface water molecules confined by the charged residues in a pit in the apo state and the typical ultrafast relaxation of 30 water molecules in the outer hydration layers of the protein surface in the football state. The subsequent local collective water relaxations and correlated water rearrangements with the protein seem as usual to be within a few and tens of picoseconds, respectively.

We previously observed the coupled water–protein relaxations on the picosecond time scales. After we obtained the water dynamics for the four systems above, we further examined the wobbling motions of the probe. Figure 2D shows the anisotropy dynamics of the F281W mutant in the apo state. Within 3 ns, we performed femtosecond-resolved fluorescence measurements of the parallel (*I*_∥_) and perpendicular (*I*_⊥_) transients. Outside that time window, we employed a time-correlated single-photon counting (TCSPC) system with an instrument response of ∼600 ps and a time window of 100 ns. The resulting anisotropy, *r*(*t*), is shown in the Supporting Information. The femtosecond-resolved anisotropy is modeled as a multiexponential decay with four components: τ_IC_, τ_W2_, τ_W3_, and τ_T_. The fastest decay, τ_IC_, is associated with the internal conversion between the nearly degenerate tryptophan excited-state ^1^L_b_ to ^1^L_a_, through conical intersection, which occurs in less than 100 fs.^43^ The slowest decay τ_T_ is associated with the tumbling of the entire protein system, which is significantly longer than the tryptophan lifetime on the nanosecond time scale, and this component is thus constant in our entire time window. The long-time range of the TCSPC anisotropy measurement allows for a more precise measurement of the amplitude associated with τ_T_. Corresponding to the observed two picosecond solvation times, here we also observed two wobbling times of τ_2W_ and τ_3W_, indicating the coupled motions between the local protein and hydration water. We then calculated the angular speed of the wobbling motion, defined as  (*i* = 2, 3), where θ_*i*_ is the wobbling cone semiangle calculated in the Supporting Information. The protein side-chain motions were measured to be 27 ps (0.71°/ps) and 699 ps (0.0269°/ps) for apo F44W, and 21 ps (1.03°/ps) and 904 ps (0.0237°/ps) for football F44W. For F281W, the motions were measured to be 12 ps (0.81°/ps) and 2.0 ns (0.0077°/ps) for the apo state, and 11 ps (1.57°/ps) and 524 ps (0.0392°/ps) for the football state. The first wobbling speed, ω_2_, is consistent with the values previously reported for other systems but the second wobbling speed, ω_3_, is substantially slower compared with those in other systems.^34−37^ This observation can be rationalized because the second wobbling (τ_3W_) is related to the local protein structural integrity, and thus, the time (τ_3W_) is much longer than that of a single globular protein, leading to the small speed of the slow relaxation.

Similarly, we also calculated the solvation speed as defined by , where Δ*E*_*i*_ is the solvation energy in cm^–1^ obtained from the solvation percentage and the total solvation stabilization energy. This speed provides a description of the rate of energy relaxation for each process (see Table 1). Overall, the *S*_1_ (∼500 cm^–1^/ps) for F44W (apo and football) and F281W (apo) is slow by a factor of 3 compared with the values of football F281W as well as the other proteins.^34−36^ The slowdown is due to the unique cavity water (football F44W), the confinement of water molecules in the cave (apo F44W), and the exterior facing charge-surrounded pit (apo F281W). The *S*_1_ (∼1500 cm^–1^/ps) for football F281W is similar to those of the outer-layer hydration water on the protein surface. We plotted the second and third solvation speeds with the corresponding two angular speeds in Figure 4, along with the data for two proteins, rat liver fatty acid-binding protein (rLFABP)^34^ and γM7-Crystallin,^35^ for comparison. Clearly, *S*_2_ and ω_2_ are relatively smaller for F44W (apo and football) and F281W (apo) but are typical for F281W (football) for a reason similar to that for *S*_1_ above. From our previous temperature studies,^36,44,45^ on the picosecond time scales, the wobbling motions of the probe could be mostly driven by the hydration water relaxations. The slow water (*S*_2_) also results in the slow wobbling (ω_2_) of the probe. For the long solvation and wobbling, clearly the ω_3_ is significantly smaller than most of the values of the other two proteins,^34,35^ resulting from the local architectures of the large complex and slow motions coupled with the water rearrangements. These comparisons are consistent with their hydration dynamics and the nature of the giant complex.

**Figure 4:**
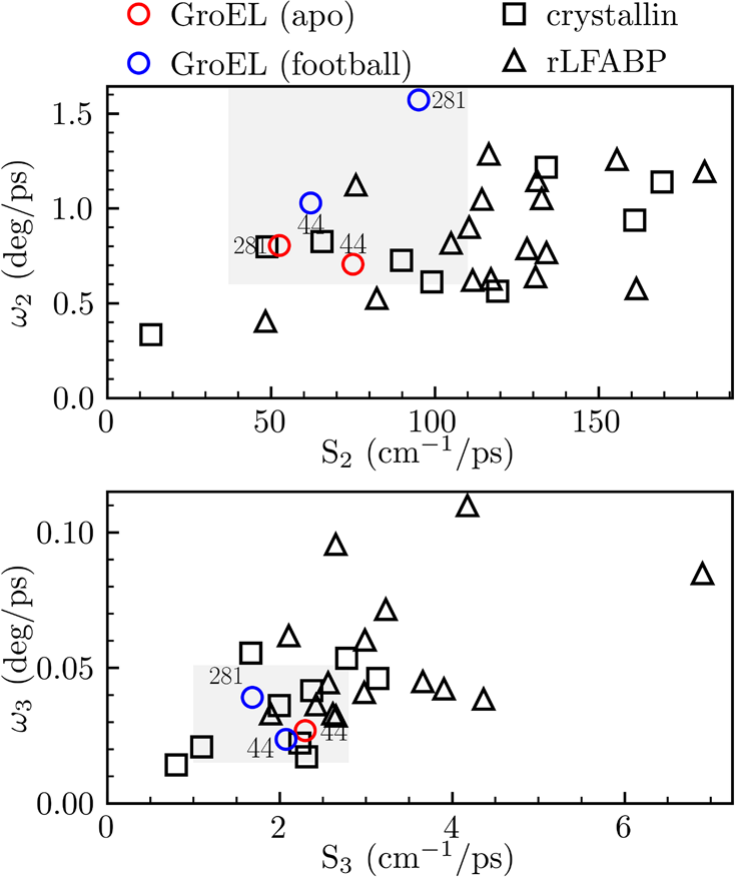
Correlations of anisotropy speed (ω) and solvation speed (*S*) for the components in a few and tens of picoseconds. The values are compared with measurements from two previous systems, Crystallin (square) and rLFABP (triangle). The results in this Letter are represented as red (apo) and blue (football) circles. Note that F281W apo is not shown on the bottom plot because of quenching at this site (Table 1).

The water dynamics at two mutation sites in the apical and equatorial domains, respectively, of apo (GroEL_14_) and football (GroEL_14_:GroES_14_) complexes were studied here using intrinsic tryptophan as an optical probe with femtosecond resolution. Drastically different initial water dynamics were observed around the two sites in the two states, reflecting their various local water dynamical and structural properties. Because of the seven GroEL subunits that form each ring, we actually probed two different water regions (Figure 5): one is a continuous water ring sensed by the equatorial F44W mutant with the inner and outer dimeters of 2.9 and 7.1 nm for the apo and 2.7 and 7.4 nm for the football states, respectively. Each ring is a hydration belt near the protein surface of the equatorial domain. The F281W mutant in the apo state probes seven water clusters on the exterior of GroEL arranged in a large ring with an inner diameter of 5.8 nm and outer diameter of 11.7 nm. In the football state, the F281W mutant probes a ring of seven water clusters inside the cavity with inner and outer diameters of 4.1 and 9.5 nm, respectively.

**Figure 5:**
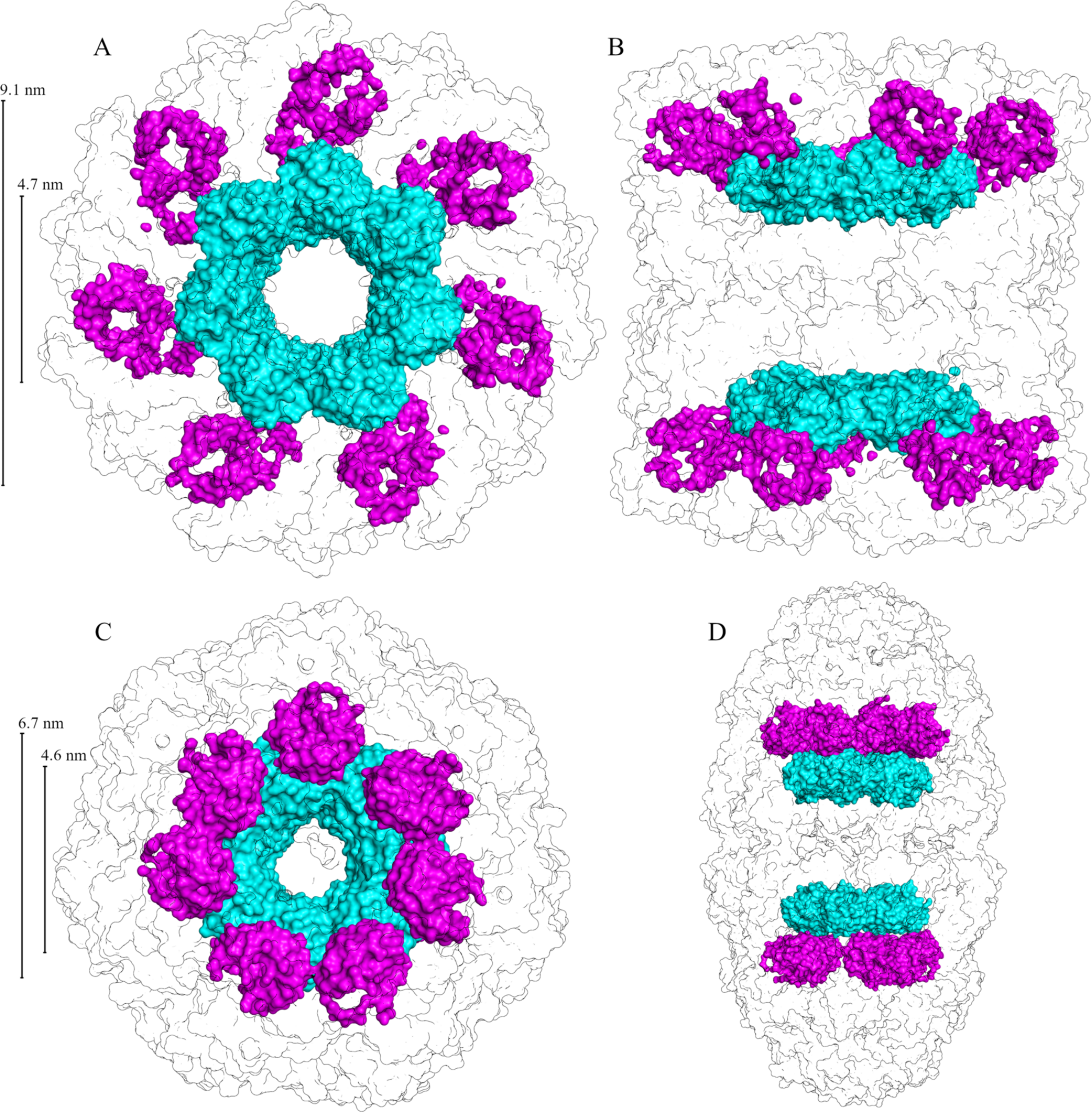
Surface representation of local water probed by tryptophan with protein outline. The top row [A and B] shows the apo state, and the bottom row [C and D] shows the football state. The left column [A and C] shows a top view from the protein exterior, and the right column [B and D] shows a 90° rotation side view. Water probed by the F44W mutant is colored in cyan, and water probed by the F281W mutant is shown in pink.

Specifically, for the F44W mutant, we observed the slowdown of the initial ultrafast water dynamics to about 1 ps, slower than the typical decay in ∼500 fs for the bulk-type water molecules in the outer hydration layers. The slowing may indicate a more structured water network, similar to the water dynamics near the lipid heads in a cubic lipidic phase which also consists of various cavities^38^ with a more rigid water structure. For the apo F44W site, more water molecules are confined in the cave and their motions are hindered with less mobility. When GroEL undergoes significant structural changes to reach the football state, the environment near the F44W probe becomes less crowded as the local environment is reconfigured and the GroEL cavity volume increases. Even though the probe is fully exposed, we found that the bulk-type water in the outer hydration layers in the cavity slows down, indicating a more rigid water network or a preferred alignment of water molecules induced by the highly charged local environment. For apo F281W, all probed water molecules are located near the protein surface, but they show an ultrafast motion in 500 fs, indicating highly frustrated motions in a small pit surrounded by charged residues. These motions are different from the typical protein surface water dynamics that occur in a few picoseconds. Similarly, in the football state, F281W undergoes a significant structural change and instead faces toward the main cavity. The probe is now fully exposed to cavity water and the outer-layer hydration water shows a typically bulk-type relaxation in ∼500 fs.

The long-time collective relaxation in a few picoseconds and cooperative rearrangement in tens of picoseconds are similar to those of surface water of globular proteins. Similarly, the hydration water couples with the local protein fluctuations. Clearly, the initial coupled dynamics of water–protein interactions in a few picoseconds are similar to those on the globular protein surface with relatively slow relaxation due to the initial water slowdown. In the long-time relaxation, because of the large complex, the local protein experiences a significantly slower motion than the surface water. In summary, the two rings in each state show different initial water dynamics with more rigid water networks in the equatorial domain and more flexible water architectures in the apical domain. The surface water–protein coupled relaxations are similar for the two mutants in the two domains. The observation of the different dynamics of cavity water, with a slowdown from the apical to equatorial domain, shows a unique hydration pattern in the GroEL cavity and may provide evidence for a water-mediated GroEL mechanism for substrate folding. Further experiments are required to understand the extent to which the water-mediated effects influence the systems function. Future work will take advantage of the methodology described in this work to characterize additional sites in the cavity also in the presence of encapsulated substrates.

## Supporting Information Available

Full sample preparation, detailed information on the fitting results of hydration water dynamics and tryptophan side-chain relaxations, and supplementary figures of anisotropy dynamics (PDF)
