# Supplementary material for: Slowdown of Water Dynamics from the Top to the Bottom of the GroEL Cavity
Source: J Phys Chem Lett. 2021 Jun 15;12(24):5723–30. doi: 10.1021/acs.jpclett.1c01216 (PMC8687601; doi:10.1021/acs.jpclett.1c01216)
Supplement: Supplementary file 1 — jz1c01216_si_001.pdf [file jz1c01216_si_001.pdf]

Supplementary Information for

Water Dynamics in the Chaperone GroEL Cavity

Nicolas Macro[a], Long Chen[a], Yushan Yang[a], Tridib Mondal[b], Lijuan Wang[a], Amnon Horovitz[*,b], Dongping Zhong[a,*]

[a]*Department of Physics, Department of Chemistry and Biochemistry, Programs of Biophysics, Program of Chemical Physics, and Program of Biochemistry, The Ohio State University, Columbus, Ohio 43210, USA.*

[b]*Department of Structural Biology, Weizmann Institute of Science, Rehovot 76100, Israel.*

[*]To whom correspondence may be addressed.
Dongping Zhong **Email:** zhong.28@osu.edu
Amnon Horovitz **Email:** amnon.horovitz@weizmann.ac.il

This PDF file includes:

Supplementary text
Figures S1 to S4
SI References

SI Materials and Methods

Molecular Biology. The F44W mutation was introduced into the gene coding for wild-type GroEL in the pOA plasmid as described.[1] The mutation F281W was introduced into this gene using restriction-free (RF) cloning[2] and the primers:
For:
5'- CGCTGCGGTTAAAGCACCGGGCTGGGGCGATCGTCGTAAAGCTATGCTG -3
Back: 5'-CATGCCGCCCATGCCACCCATG-3'. The mutations were confirmed by DNA sequencing of the entire genes.

GroEL Purification. Both GroEL and GroES were produced using DH5α *E. coli* cells. The cells were grown using 2xYT medium with 100 μg/mL ampicillin at 37 °C with shaking at 220 RPM. Cells are grown for 24 hours with a second dose of 50 μg/mL ampicillin after 9 hours of growth.[3]

To purify GroEL, cells are lysed using sonication on ice and centrifuged to remove cell lysate. The supernatant is then precipitated using 55% ammonium sulfate. The precipitant is then resuspended and applied to a Q anion exchange column (Bio-Rad) with elution using a NaCl gradient to 1 M final concentration. Fractions of 5 mL volume are collected and only peak fractions with steady-state absorbance A_{260} / A_{280} < 1 are combined and precipitated with 55% ammonium sulfate. The precipitant is then resuspended and applied to a SDG25 column and subsequently concentrated and applied to a Superose 6 column (GE Lifesciences) with an ATP $MgCl_2$ buffer. The peak protein is then precipitated using 55% ammonium sulfate. The precipitant is then resuspended and applied to a Q anion exchange column (Bio-Rad) with a methanol buffer with elution using a NaCl gradient to 1 M final concentration. Peak fractions are combined and applied to an SDG column and the peak fractions are diluted and precipitated using 45% acetone.[4] Dry and resuspend the precipitant using G10K buffer (50 mM Tris (pH 7.5), 10 mM KCl, 10 mM $MgCl_2$, 1 mM DTT). All apo measurements were performed with protein in G10K buffer. GroEL purity was evaluated by performing steady-state emission measurements on WT GroEL, which contains no tryptophan, with an excitation at 295 nm. The contaminant tryptophan concentration was measured to be 0.02 mol contaminant tryptophan per mol GroEL subunit. The entire GroEL purification was performed at room temperature. GroEL in the apo state was stored in G10K Buffer (10 mM KCl, 10 mM $MgCl_2$, 1 mM DTT) at -80 °C.

GroES Purification. To purify GroES, cells are lysed using sonication on ice and centrifuged at 20,000 RPM and 4 °C for 30 minutes to remove cell lysate. The supernatant is then placed in a 60 °C heat bath for 10 minutes. The mixture is then centrifuged at 20,000 RPM and 4 °C for 30 minutes, and the supernatant is applied to a Ni-NTA column (Bio-Rad). Once the protein has been loaded into the column, it is washed with five column volumes (CV) Buffer A (50 mM Tris-HCl (pH 7.5), 500 mM NaCl, 10 mM β-Mercaptoethanol, 10 mM imidazole) followed by seven CV Buffer A with 2 mM ATP and 10 mM $MgCl_2$ and finally 5 CV Buffer A. An imidazole gradient (10 mM imidazole – 500 mM imidazole) is used to elute GroES.[5] The sample is dialyzed against G10K Buffer. The entire GroES purification was performed at 4 °C. GroES was stored in G10K Buffer at -80 °C. The complete protocol is detailed in Supporting Information. Briefly, the GroEL footballs are produced by combining GroEL with excess GroES in an Activation Buffer

(16.7 mM ATP (pH 8.0), 167 mM Na_2SO_4, 167 mM NaF, 16.7 mM $BeSO_4$, 5 mM DTT).[6] The solution is then applied to a Superose 6 column (GE Lifesciences) to remove the excess GroES. Measurements in the football state were performed in HKM-Be Buffer (20 mM HEPES (pH 7.5), 100 mM KCl, 50 mM $MgCl_2$, 50 mM Na_2SO_4).

Football Formation. To construct footballs state GroEL, 1.1 μM GroEL and 4.4 μM GroES are combined along with 70 mM $MgCl_2$ and 140 mM KCl. The protein solution is then diluted using Folding Buffer (50 mM Tris (pH 7.5), 140 mM KCl, 70 mM $MgCl_2$, and 5 mM DTT) to a final volume of 20 mL. Once the diluted protein solution has been stirred for 10 minutes, 7.3 mL of Activation Buffer (16.7 mM ATP (pH 8.0), 167 mM Na_2SO_4, 167 mM NaF, 16.7 mM $BeSO_4$, 5 mM DTT) is added.[7] The solution is then stirred for 10 minutes and allowed to sit without stirring for 35 minutes. Sample is centrifuged and the supernatant is concentrated and applied to a Superose 6 column (GE Lifesciences) prepared with HKM-Be Buffer (20 mM HEPES (pH 7.5), 100 mM KCl, 50 mM $MgCl_2$, 50 mM Na_2SO_4). The entire football construction process was performed at room temperature. Measurements in the football state were performed in HKM-Be Buffer (20 mM HEPES (pH 7.5), 100 mM KCl, 50 mM $MgCl_2$, 50 mM Na_2SO_4).

Upconversion measurements were performed with sample concentrations between 300-1000 μM. Time correlated single photon counting measurements were performed using sample concentrations between 100-300 μM. Steady-state emission measurements were performed at 5-15 μM.

Transmission Electron Microscopy Measurement. GroEL sample was diluted to ~10 nM and placed on a glow discharged carbon coated copper grid. A 2% uranyl acetate was used to negatively stain the samples.[8] Measurements were taken with a Tecnai G2 Spirit (FEI) with 120 kV accelerating voltage.

Femtosecond-Resolved Fluorescence Spectroscopy. We excited the GroEL sample with a ~120 nJ pulse energy at 293 nm central wavelength focused onto a rotating sample cell. The fluorescence is then collected using off-axis parabolic mirrors and is then focused onto a 0.2 mm β-barium borate (BBO) crystal where it is mixed with an 800 nm gating beam to generate the upconverted signal. The upconverted signal was then detected using a photomultiplier tube through a monochromator. The signal from the photomultiplier tube is digitized using a boxcar average. This provided us with a cross correlation width of 400 – 500 fs, as measured using water scattering collected at 320 nm. The solvation measurements used a pump polarization at the magic angle (54.7°) with respect to the acceptance angle. Anisotropy measurements were performed with parallel and perpendicular polarizations relative to the acceptance direction of the gating BBO crystal. The full details of the experimental method have been described previously.[9]

Sub-nanosecond-resolved Time Correlated Single Photon Counting. Measurements were performed using a commercially available FluoTime 200 system (PicoQuant) with excitation from a PLS-290 pulsed LED (8 MHz, 290 nm, ~1 μW, PicoQuant). The instrument response function was measured to be ~600 ps with a time window of 100 ns. Anisotropy measurements were taken at with all four combinations of horizontal and

vertical pump and detection polarization to calculate and compensate for the detector's polarization bias. These data were analyzed using FluoFit (PicoQuant).

Molecular Dynamics Simulation. We performed all simulations using the GROMACS 2018.2 using the CHARMM36-november18 force field.[10] We obtained crystal structures from the Protein Data Bank using structure IDs 5DA8 and 4PKO for the GroEL apo and football states, respectively. Prior to simulation and mutation, all heteroatoms were removed from the crystal structures. We used PyMol to mutate the probe site to tryptophan on each of the 14 monomers, using the lowest energy state for the mutant. The protein was solvated using TIP3P water and neutralized by adding 266 and 293 Na^+ ions for the apo and football states, respectively. The systems were initially run through an energy minimization using the steepest descent algorithm with a step size of 1 Å until the maximum force was smaller than 1000 kJ/mol/nm. The temperature was raised to 300 K using a 100 ps simulation with a step time of 2 fs and a constant box volume with periodic boundary conditions while constraining bonds with hydrogen atoms using the LINCS algorithm. Subsequently, it was relaxed to a pressure of 1 bar for 100 ps with a step size of 2 fs. A three nanosecond production simulation was performed for all systems. All analysis was performed using MDAnalysis after a one-nanosecond burn-in truncation.[11,12]

Data Analysis. The femtosecond-resolved transients are fit using a multiexponential decay model,

$$I_\lambda(t) = I_\lambda^{solv}(t) + I_\lambda^{pop}(t) = \sum_i a_i e^{-\frac{t}{\tau_i}} + \sum_j b_j e^{-\frac{t}{\tau_j}} \quad (1)$$

The first term contains components associated with solvation processes, while the second term contains the lifetime associated decays. For fluorescence measured red of the steady-state emission peak, the solvation component amplitude, a_i, are negative. The lifetime decay amplitudes b_j are positive for all wavelengths. The lifetime decay times τ_j are the same for all wavelengths. The overall emission spectrum can be computed as,

$$I(\lambda, t) = \frac{I_{SS}(\lambda) I_\lambda(t)}{\sum_i a_i \tau_i + \sum_j b_j \tau_j} \quad (2)$$

where $I_{ss}(\lambda)$ is the steady-state emission spectrum. Additionally, the lifetime associated spectrum can be computed as,

$$I^{pop}(\lambda, t) = \frac{I_{SS}(\lambda) I_\lambda^{pop}(t)}{\sum_i a_i \tau_i + \sum_j b_j \tau_j} \quad (3)$$

These spectra are fit with a lognormal model and the time-resolved peak maxima ($\nu_s(t)$ and $\nu_l(t)$) are generated. Using these peak maxima, we can construct the correlation function,

$$c(t) = \frac{\nu_t(t) - \nu_l(t)}{\nu_t(0) - \nu_l(0)} \quad (4)$$

$$c(t) = A_1 e^{-\frac{t}{\tau_{1s}}} + A_2 e^{-\frac{t}{\tau_{2s}}} + A_3 e^{-\frac{t}{\tau_{3s}}}, \quad \sum_{i=1}^{3} A_i = 1 \tag{5}$$

The correlation function is then fit with a multiexponential decay model, which provides the amplitude, A_i, and decay time, τ_i. The total Stokes shift, $\Delta E_{total} = \nu_t(0) - \nu_l(0)$, is used with the amplitudes to produce the solvation speed,

$$\Delta E_i = \Delta E_{total} A_i \tag{6}$$

$$S_i = \frac{\Delta \mathrm{E}_i}{\tau_i} \tag{7}$$

The anisotropy is calculated using

$$r(t) = \frac{I_{\parallel}(t) - I_{\perp}(t)}{I_{\parallel}(t) + 2I_{\perp}(t)} \tag{8}$$

The resulting trace is then fit with a multiexponential decay model

$$r(t) = r_{IC} e^{-\frac{t}{\tau_{IC}}} + r_2 e^{-\frac{t}{\tau_{2W}}} + r_3 e^{-\frac{t}{\tau_{3W}}} + r_T \tag{9}$$

The first component (IC) typically has a decay time less than 100 fs after deconvolution from the instrument response and is attributed to the internal conversion between the ^1L_b and ^1L_a excited states.
The wobbling semiangle is estimated as

$$1 - \frac{r_{iW}}{\sum_j r_{jW} + r_T} = \left(\frac{3\cos^2\theta_i - 1}{2}\right)^2 \quad i, j = 2, 3 \; j \leq i \tag{10}$$

The wobbling angular speed is defined as

$$\omega_i = \frac{\theta_i}{\tau_{iW}} \quad i = 2, 3 \tag{11}$$

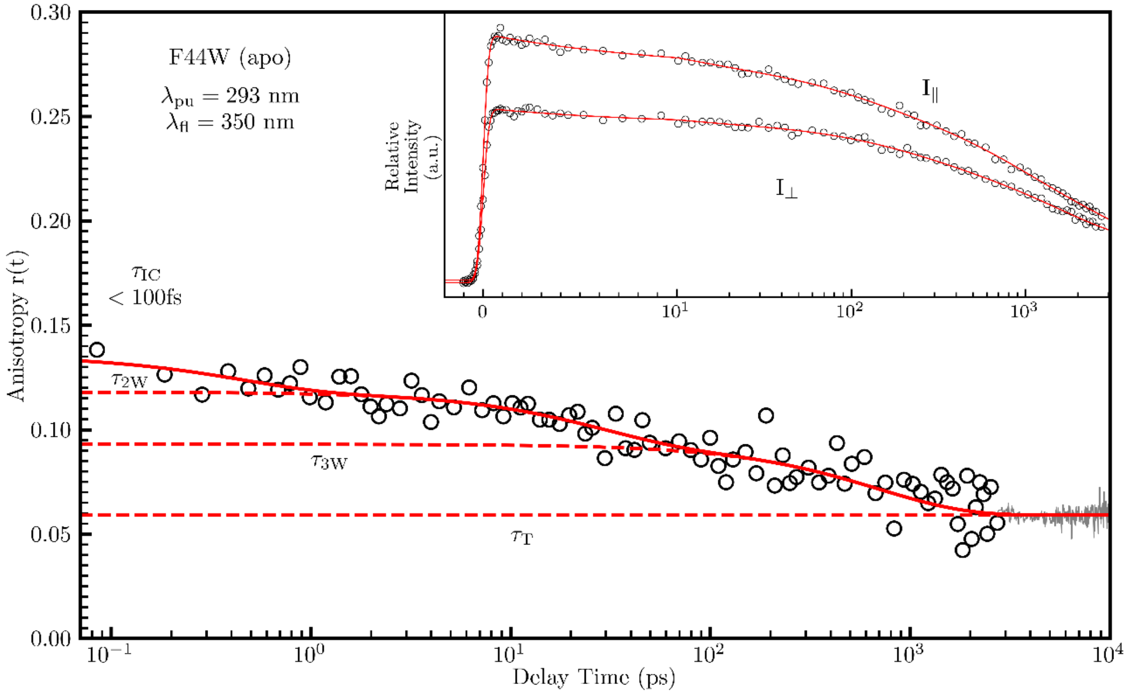

Fig. S1. Apo F44W upconversion anisotropy data (circles), long-time TCSPC data (gray), and fit line (red) with associated upconversion transients in inset.

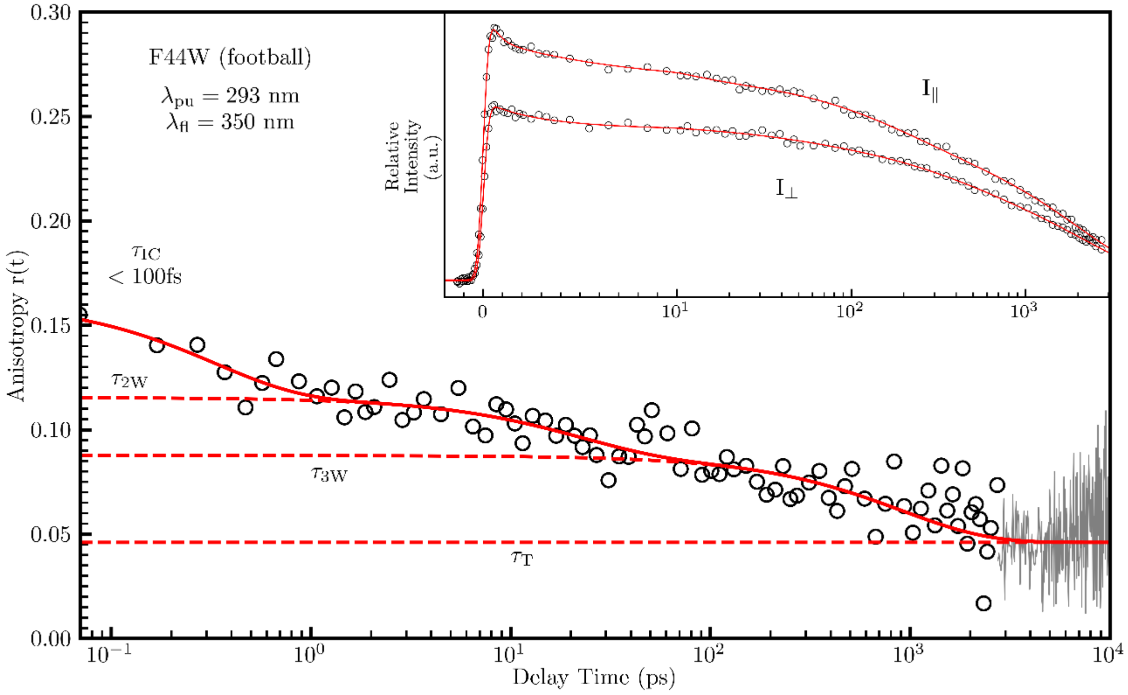

Fig. S2. Football F44W upconversion anisotropy data (circles), long-time TCSPC data (gray), and fit line (red) with associated upconversion transients in inset.

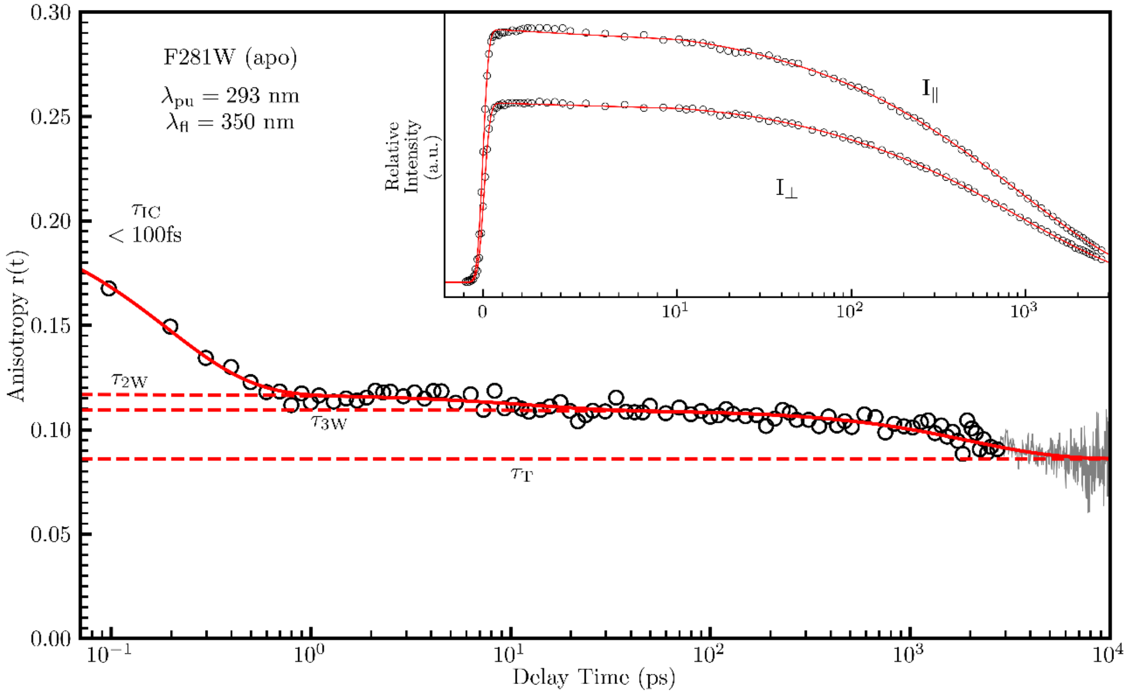

Fig. S3. Apo F281W upconversion anisotropy data (circles), long-time TCSPC data (gray), and fit line (red) with associated upconversion transients in inset.

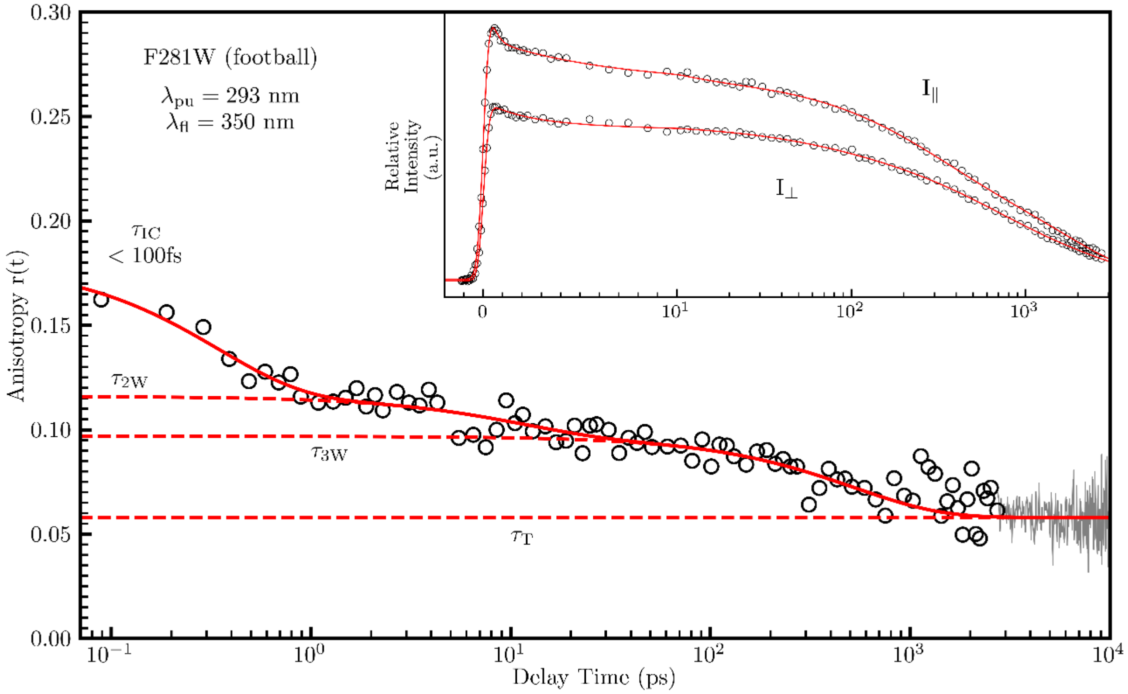

Fig. S4. Football F281W upconversion anisotropy data (circles), long-time TCSPC data (gray), and fit line (red) with associated upconversion transients in inset.

SI References

(1) Yifrach, O.; Horovitz, A. Transient Kinetic Analysis of Adenosine 5'-Triphosphate Binding-Induced Conformational Changes in the Allosteric Chaperonin GroEL. *Biochemistry* **1998**, *37* (20), 7083–7088. https://doi.org/10.1021/bi980370o.

(2) Unger, T.; Jacobovitch, Y.; Dantes, A.; Bernheim, R.; Peleg, Y. Applications of the Restriction Free (RF) Cloning Procedure for Molecular Manipulations and Protein Expression. *Journal of Structural Biology* **2010**, *172* (1), 34–44. https://doi.org/10.1016/j.jsb.2010.06.016.

(3) Dyachenko, A.; Gruber, R.; Shimon, L.; Horovitz, A.; Sharon, M. Allosteric Mechanisms Can Be Distinguished Using Structural Mass Spectrometry. *PNAS* **2013**, *110* (18), 7235–7239. https://doi.org/10.1073/pnas.1302395110.

(4) Dyachenko, A.; Gruber, R.; Shimon, L.; Horovitz, A.; Sharon, M. Allosteric Mechanisms Can Be Distinguished Using Structural Mass Spectrometry. *PNAS* **2013**, *110* (18), 7235–7239. https://doi.org/10.1073/pnas.1302395110.

(5) Franck, J. M.; Sokolovski, M.; Kessler, N.; Matalon, E.; Gordon-Grossman, M.; Han, S.; Goldfarb, D.; Horovitz, A. Probing Water Density and Dynamics in the Chaperonin GroEL Cavity. *J. Am. Chem. Soc.* **2014**, *136* (26), 9396–9403. https://doi.org/10.1021/ja503501x.

(6) Taguchi, H.; Tsukuda, K.; Motojima, F.; Koike-Takeshita, A.; Yoshida, M. BeFx Stops the Chaperonin Cycle of GroEL-GroES and Generates a Complex with Double Folding Chambers. *J. Biol. Chem.* **2004**, *279* (44), 45737–45743. https://doi.org/10.1074/jbc.M406795200.

(7) Taguchi, H.; Tsukuda, K.; Motojima, F.; Koike-Takeshita, A.; Yoshida, M. BeFx Stops the Chaperonin Cycle of GroEL-GroES and Generates a Complex with Double Folding Chambers. *J. Biol. Chem.* **2004**, *279* (44), 45737–45743. https://doi.org/10.1074/jbc.M406795200.

(8) Harris, J. R.; Plückthun, A.; Zahn, R. Transmission Electron Microscopy of GroEL, GroES, and the Symmetrical GroEL/ES Complex. *Journal of Structural Biology* **1994**, *112* (3), 216–230. https://doi.org/10.1006/jsbi.1994.1022.

(9) Zhang, L.; Kao, Y.-T.; Qiu, W.; Wang, L.; Zhong, D. Femtosecond Studies of Tryptophan Fluorescence Dynamics in Proteins: Local Solvation and Electronic Quenching. *J. Phys. Chem. B* **2006**, *110* (37), 18097–18103. https://doi.org/10.1021/jp063025e.

(10) Abraham, M. J.; Murtola, T.; Schulz, R.; Páll, S.; Smith, J. C.; Hess, B.; Lindahl, E. GROMACS: High Performance Molecular Simulations through Multi-Level Parallelism from Laptops to Supercomputers. *SoftwareX* **2015**, *1–2*, 19–25. https://doi.org/10.1016/j.softx.2015.06.001.

(51) Gowers, R.; Linke, M.; Barnoud, J.; Reddy, T.; Melo, M.; Seyler, S.; Domański, J.; Dotson, D.; Buchoux, S.; Kenney, I.; Beckstein, O. MDAnalysis: A Python Package for the Rapid Analysis of Molecular Dynamics Simulations; Austin, Texas, 2016; pp 98–105. https://doi.org/10.25080/Majora-629e541a-00e.

(52) Michaud-Agrawal, N.; Denning, E. J.; Woolf, T. B.; Beckstein, O. MDAnalysis: A Toolkit for the Analysis of Molecular Dynamics Simulations. *Journal of Computational Chemistry* **2011**, *32* (10), 2319–2327. https://doi.org/10.1002/jcc.21787.
